# Thrombotic microangiopathies in Pediatrics: current nomenclature and differential diagnoses with Thrombocytopenia-associated multiple organ failure (TAMOF)

**DOI:** 10.1590/1984-0462/2022/40/2021156

**Published:** 2021-12-20

**Authors:** João Paulo Silva Cezar, Selma Harue Kawahara, Frederico Ribeiro Pires

**Affiliations:** a Unidade de Terapia Intensiva, Hospital da Criança de Brasília José Alencar, Brasília, DF, Brazil.

In their recent article, *Hemolytic-uremic syndrome associated with Streptococcus pneumoniae in pediatrics: a case series*, published in Revista Paulista de Pediatria, Guerra et al.^1^ reported four cases of thrombotic microangiopathy (TMA) related to pneumococcal infection in the unicentric pediatric population of Bogotá, Colombia. One can highlight the excellence in the management of these cases, which is exemplified by the absence of deaths and recovery of renal function in three of the four children. This is a rare entity in childhood, and the incidence of four cases in a short period is relevant and reinforces the importance of this service for the local population. The clear and objective description of the cases enhances the knowledge of the scientific community.

This publication suggests that TMA associated with infection by *S. pneumoniae* is reported as hemolytic-uremic syndrome (HUS) or atypical HUS, as noted by some authors, which is currently avoided because it is a generic term grouping different diseases, with different treatments, in the same classification. The current preferred trend is the subclassification of TMA by the underlying pathophysiology: mediated by ADAMTS-13 deficiency instead of thrombotic thrombocytopenic purpura; mediated by complement instead of atypical HUS; and mediated by Shiga toxin instead of typical HUS, among others.^2^

It is also worth mentioning that, in all four cases, the children developed microangiopathic hemolytic anemia and thrombocytopenia, followed by acute kidney injury and the need for renal replacement therapy. Given the presentation of thrombocytopenia associated with the failure of two or more systems, we suggest the diagnosis of thrombocytopenia-associated multiple organ failure (TAMOF), which has characteristic histological and laboratory evidence, worse outcomes, and few therapeutic possibilities.^3^ ^,^ ^4^ This diagnosis was, however, not suggested in the discussion of the article. TAMOF is probably the key diagnosis for these patients. Furthermore, pneumococcal-mediated TMA fits the diagnosis of TAMOF. Within this presentation, one can observe the described disease evolution in case three, with multiple hemorrhages and ischemic lesions of extremities, requiring amputation. This presentation can occur due to the expression of tissue factors resulting from the microvascular endothelial lesion by sepsis with consequent formation of fibrin microthrombi, in addition to maladjustment of the anticoagulant system. The phenomenon presented suggests disseminated intravascular coagulation, which we consider missing from this important differential in the cases described.

This letter is intended to highlight the brilliant article cited, alongside some rare differential diagnoses in pediatrics and the current nomenclature for thrombotic microangiopathies.
